# The Genetic Landscape of Ocular Adnexa MALT Lymphoma Reveals Frequent Aberrations in NFAT and MEF2B Signaling Pathways

**DOI:** 10.1158/2767-9764.CRC-21-0022

**Published:** 2021-10-13

**Authors:** Marco Magistri, Lanie E. Happ, Jeremy Ramdial, XiaoQing Lu, Vasileios Stathias, Kranthi Kunkalla, Nitin Agarwal, Xiaoyu Jiang, Stephan C. Schürer, Sander R. Dubovy, Jennifer R. Chapman, Francisco Vega, Sandeep Dave, Izidore S. Lossos

**Affiliations:** 1 Division of Hematology, Department of Medicine, Sylvester Comprehensive Cancer Center, University of Miami, Miami, Florida.; 2 Center for Genomic and Computational Biology and Department of Medicine, Duke University, Durham, North Carolina.; 3 Department of Molecular and Cellular Pharmacology, University of Miami Miller School of Medicine, Miami, Florida.; 4 Center for Computational Science, Sylvester Comprehensive Cancer Center, University of Miami, Miami, Florida.; 5 Division of Hematopathology, Department of Pathology and Laboratory Medicine, University of Miami, Miami, Florida.; 6 Bascom Palmer Eye Institute, University of Miami Miller School of Medicine, Miami, Florida.

## Abstract

A comprehensive constellation of somatic nonsilent mutations and copy-number (CN) variations in ocular adnexa marginal zone lymphoma (OAMZL) is unknown. By utilizing whole-exome sequencing in 69 tumors, we define the genetic landscape of OAMZL. Mutations and CN changes in CABIN1 (30%), RHOA (26%), TBL1XR1 (22%), and CREBBP (17%) and inactivation of TNFAIP3 (26%) were among the most common aberrations. Candidate cancer driver genes cluster in the B-cell receptor (BCR), NF-κB, NOTCH, and NFAT signaling pathways. One of the most commonly altered genes is *CABIN1*, a calcineurin inhibitor acting as a negative regulator of the NFAT and MEF2B transcriptional activity. CABIN1 deletions enhance BCR-stimulated NFAT and MEF2B transcriptional activity, while CABIN1 mutations enhance only MEF2B transcriptional activity by impairing binding of mSin3a to CABIN1. Our data provide an unbiased identification of genetically altered genes that may play a role in the molecular pathogenesis of OAMZL and serve as therapeutic targets.

**Significance:**

We report systematic application of whole-exome sequencing and CN variations in OAMZL, revealing common alterations in regulation of NFAT signaling pathway that may facilitate identification of new therapies.

## Introduction

The mutational landscape of many subtypes of non-Hodgkin's lymphomas (NHL) has been elucidated through multiple whole-genome, whole-exome, and targeted sequencing projects (1–7). These studies together with analyses characterizing the functional consequences of the identified mutations have greatly helped illuminate NHL pathogenesis and have translated into clinical investigations and practice (e.g., therapies such as ibrutinib and tazemetostat; refs. 8–10). However, while the majority of these studies focused on common subtypes of NHL, such as diffuse large B-cell lymphoma (DLBCL), follicular lymphoma, and Mantle cell lymphoma (MCL), the genetic alterations and functional consequences of somatic mutations in several less prevalent and less studied lymphoma subtypes remain largely unexplored.

Marginal zone lymphomas (MZL), which represent 6% to 9% of all NHLs, are subclassified into extranodal MZL (EMZL) of mucosa-associated lymphoid tissue (MALT), nodal MZL (NMZL), and splenic MZL (SMZL) based on the involved site (11). These lymphomas are assumed to originate from MZ B cells of the secondary lymphoid follicles. In humans, MZ B cells are confined to anatomic locations continuously exposed to antigens, such as the spleen, the epithelium of tonsillar crypts, and the subepithelial area of MALT, mainly in intestinal Peyer's patches and the inner wall of the subcapsular sinus of lymph nodes (12). MZ zone B cells play important roles in innate-like rapid antibody responses, mainly to T cell–independent (e.g., polysaccharides) and less frequently to T cell–dependent antigens (13).

EMZLs are the most common subtype, accounting for 50% to 70% of all MZLs (11, 14). These lymphomas originate in organs that normally lack lymphoid tissue but accumulate B cells in response to chronic inflammation from infection or autoimmune processes (14). The most common gastric EMZLs are associated with *Helicobacter pylori* infection and their pathogenesis has been extensively studied (15). The less common intestinal and skin MALT lymphomas are linked to infections with *Campylobacter jejuni* and *Borrelia burgdorferi*, respectively, while Hashimoto's thyroiditis and Sjögren syndrome are associated with EMZLs of the thyroid and salivary glands, respectively (16–19). *Chlamydophila psittaci* infection has been implicated in the pathogenesis of ocular adnexal MZLs (OAMZLs), the second most common EMZL and the most common tumor occurring in the ocular adnexa (20). However, this association demonstrated marked differences based on geography and was not observed in most North American studies (21).

Analysis of immunoglobulin (Ig) gene usage and mutation patterns showed restricted immunoglobulin gene repertoire and mutations, suggesting these tumors have undergone affinity maturation (22, 23). The demonstrated polyreactivity of these Igs to self-antigens implicates B-cell receptor (BCR) stimulation in the pathogenesis of these lymphomas (22, 24). However, intrinsic genetic aberrations contributing to the pathogenesis of OAMZLs are largely unknown. Trisomies of chromosomes 3 and 18 and deletions of 6q23 are observed in OAMZLs, as in all other MZLs, suggesting a common shared genetic aberration (16). However, the classical EMZLs chromosomal translocations t(11;18)(q21;q21) *BIRC3-MALT1*, t(1;14)(p22;q32) *BCL10-IGH* and t(14;18)(q32;q21) *IGH-MALT1*, which cause constitutive NF-κB activation, are seen primarily in EMZLs of the stomach and lung but rarely or not at all in OAMZLs (25, 26).

Marked advances in the understanding of the pathobiology of many lymphoma subtypes were achieved by the application of gene expression arrays, high-resolution SNP arrays and next-generation sequencing. Such studies markedly advanced our understanding of the pathogenesis of SMZLs and nodal MZLs (2, 27, 28). However, only two studies performed whole-genome sequencing of OAMZL (*n* = 16; refs. 29, 30) and several studies used whole-exome sequencing (WES) in 8 patients and targeted sequencing approaches to identify mutated genes, leading to the identification of recurrent mutations in *TNFAIP3*, *TBL1XR1*, *CREBBP*, *KMT2D*, *MYD88*, *NOTCH1/2*, among others (30–36). While some of these mutations were observed repeatedly in different studies, others were not. Furthermore, the mutation frequency of the genes varied significantly between studies. Because WES was done in only 8 cases, the comprehensive mutational landscape of OAMZL remains largely uncharacterized. Herein, we performed WES and present integrated results of genetic variants and copy-number (CN) alterations in 69 OAMZL cases that (i) establish the mutation landscape in these tumors; (ii) identify multiple novel genes that are recurrently mutated, deleted or gained, and (iii) characterize novel signaling pathways that are deregulated in OAMZLs and can be therapeutically targeted.

## Materials and Methods

### Patient and Control Samples

OAMZL tumors were obtained during routine diagnostic biopsies in 72 patients over 16 years. Three tumors were excluded due to inadequate mean coverage resulting in 69 tumors that originated from conjunctiva (27), lacrimal gland (7) and orbital tissue (33), and concomitant orbital tissue and lacrimal gland (2). All diagnostic specimens were reviewed by expert hematopathologists (J.R. Chapman and F. Vega) using the morphologic and immunophenotypic diagnosis of MZL defined by the WHO classification (11). All the OAMZL specimens included in this study showed B-cell monoclonality by PCR using BIOMED-2 primers (37, 38). In 7 patients (including one with inadequate mean coverage), normal tissues were obtained from blood cells simultaneously with the diagnostic biopsy. In all the specimens, the DNA was extracted using the DNeasy blood and tissue kit (QIAGEN) from fresh tumor cells and stored at −80°C. Patient tumor and normal samples, as well as clinical data, were collected following written informed consent from the patients, according to a protocol approved by the University of Miami Institutional Review Board in accordance with the Declaration of Helsinki.

### Library Preparation and WES

Genomic DNA was sheared to 250 bp using the Covaris S2 platform and then subjected to Agilent SureSelectXT2 protocol as previously described (3). Library sequencing was performed to an average of 65× coverage on Illumina Hiseq 2500 platform.

### Exome Sequence Alignment and Sample Quality Control

Reads in fastq format were processed and aligned as previously described (3). We used the same alignment pipeline as reported by Reddy and colleagues. Briefly, Illumina adapter sequences were removed using GATK (39) version 3.2 and reads were mapped to hg19 using Burrows-Wheeler Aligner (40). Depth and breadth of coverage for each exome were computed using BEDTools (41). Quality control metrics for all sequenced samples are reported in Supplementary Table S1. Three tumor samples (including one with paired normal tissue) were excluded from analysis due to inadequate mean coverage. The final data set for analysis contains tissue from 69 tumor and 7 normal samples comprising six paired tumor–normal sets.

### Variant Calling, Annotation, Filtering, and Identification of Driver Genes

GATK HaplotypeCaller (GATK version 3.2) was used for joint variant calling on all tumor and normal samples (39). This resulted in a total of 624,468 variants. All variants were annotated with gene names, predicted function, population frequencies, and other variant annotations using Annovar (42). Variant filtering was performed using the following criteria: include exonic, not synonymous or nonframeshift variants, rare variants (maximum population frequency < 0.1%), and damaging variants (CADD score > 10); exclude frameshifts found in more than one sample, variants found in genomic superduplication regions, variants found in any of the seven normal samples, and those found in highly polymorphic genes that are likely to harbor false positives in WES data, as reported previously (43, 44). In addition, each variant was required to have a read depth of at least 5. This resulted in a final set of 9,666 variants in 6,078 genes across the 69 tumor samples.

The number of HaplotypeCaller variants that passed filtering per sample ranged from a low of 32 to a high of 885 with an average of 140 (Supplementary Fig. S1). The most highly mutated sample had such a significantly high number of variants (statistical outlier by the Grubbs test, *P* < 2.2e-16) that we decided to exclude this sample from the *t* test calculation described below. This sample was not an outlier in the number of variants present in driver genes (number of driver variants in outlier sample equals 23; range of driver variants across all samples is 4–53), and subsequently was included in all further analyses.

The difference in average variants per sample for samples with paired normal (*n* = 6, mean = 71.6) and no paired normal (*n* = 62, mean = 134.7) is significant (*t* test, *P* = 0.02). Our filtering strategy will completely remove private germline variants from samples with paired normals, so this is an expected result. While private germline variants may remain in our filtered calls from samples with no paired normal, they are unlikely to cluster in cancer driver genes.

For the six tumor samples with paired normal tissue, MuTect version 1.1.4 was used to detect truly somatic single nucleotide variants (45). The original MuTect output contained 72,885 variants across the six paired samples. Annovar annotations and filtering were applied as described above. 5,613 variants across 4,011 genes passed these filtering criteria, with a range of 291 to 2,752 variants per sample (median = 353). When comparing the variant calls for samples with paired normals, we observed much higher sensitivity in variant calls from MuTect compared with GATK HaplotypeCaller, as expected. When variants were found by HaplotypeCaller in these samples, they were generally also reported by MuTect (range of concordance: 60.5%–94.7%).

**FIGURE 1:**
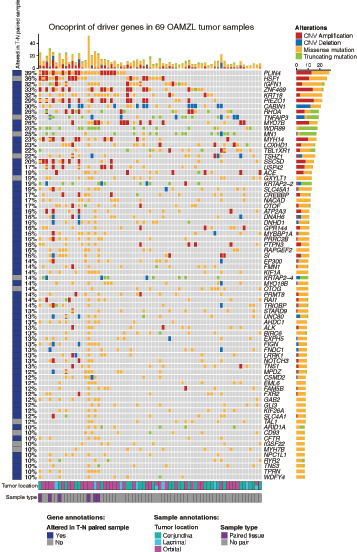
Genetic landscape of OAMZL. An oncoprint showing the mutation and CNV status for the 74 most recurrently altered genes in 69 OAMZL tumors with frequency ≥10%. Each alteration type is color coded, as indicated in the figure. Each column corresponds to one sample and includes annotations at the bottom identifying anatomic tumor location as well as those samples with paired normal tissue. Genes are represented in the rows and are annotated on the basis of whether any reported mutations are identified in at least one of the tumor sample with paired normal tissue by Mutect. In addition, we report the frequency of mutations per gene, number and composition of mutation type per gene, and number and composition of mutation type per sample.

Filtered variants from HaplotypeCaller and Mutect were aggregated by gene. Highly polymorphic genes likely to harbor false-positive variants in WES data were excluded from our driver gene analysis (43, 44). All other genes with a mutational frequency greater than 10% (mutated in at least 7 samples) were retained for further investigation as driver genes (*n* = 74 genes; Supplementary Table S2; Supplementary Fig. S2). The mutation calls from this set of recurrently mutated genes were combined with CN calls and are presented in Fig. 1. Only 6 of these 74 genes contained variants exclusively identified in samples with no paired normal tissue (*CD93*, *IGSF22*, *KRTAP2–4*, *MN1*, *MYH7B*, and *OTOG*) and none are altered at a high enough frequency for it to be statistically significant that they were not altered in the set of samples with paired normal (by Fisher exact test, maximum *P* = 0.3239). Therefore, our variant filtering and driver gene identification strategies are robust for both samples with and without paired normal tissue.

In addition to the driver genes identified for inclusion in Fig. 1, we further analyzed mutations in genes belonging to specific pathways, including BCR signaling, NF-κB signaling, NOTCH signaling, and NFAT signaling. All genes from these pathways were examined, even those with mutations in less than 10% of samples that were excluded from driver gene analysis. When mutation data are combined with CN for these genes, it is possible that their overall alteration frequency exceeds 10%.

### CN Alteration Analysis

EXCAVATOR (46) was run on each sequenced exome (including all 69 tumors and 7 normal samples) in pooled mode against the seven normal samples. This program evaluates read depths at each exon in each exome-sequencing sample and performs normalization based on the depths in the pooled normal samples. The paired normal samples were run against the pool of normal to control for biological and technical artifacts. In addition, all calls were further filtered against CN calls from a panel of 25 normal samples. The output of EXCAVATOR was provided to GISTIC 2.0 (47) to determine recurrent arm-level and broad and focal gene–level CN changes found in the OAMZL tumor dataset. Recurrent tumor alterations that were found in any of the normal samples were discarded from further analysis. Annotated raw output from this CN analysis is reported in Supplementary Table S3. Both broad and focal gene–level events were used for downstream analysis.

### Mutational Signature Analysis

The Palimpsest R package was used to extract Catalogue Of Somatic Mutations In Cancer (COSMIC) mutational signatures from the somatic driver gene variants of the OAMZL samples following previously reported approaches (48–50). The number and proportion of mutations attributed to each signature in each tumor was calculated using the single-base substitution signatures previously identified in human cancer that were not attributed to potential sequencing artifact (51). Signatures and their proposed etiology that were included in this analysis can be found in Supplementary Table S1. Associations of these signatures with driver genes were performed by assigning the most likely causative mutational signature to each individual mutation. The 12 COSMIC mutational signatures with the most associated mutations were used as input for the mutational signatures’ exposure analysis and the resulting signature probabilities for each individual mutation were then summarized to the gene level. The signature exposure profiles for the top 20 genes with the most somatic mutations (excluding CNV calls) are presented.

### RNA Extraction, RNA-Sequencing Library Preparation, and Pathway Analysis

CABIN1 knock down (KD) and CABIN1 wild-type (WT) SSK41 cells were placed in serum-free media with and without 4 hours of stimulation with 20 μg/mL of α-IgM F(ab’)_2_ (Thermo Fisher Scientific). RNA was extracted and purified using a combination of TRIzol reagent and QIAGEN RNeasy columns as described previously (52). RNA (1 μg) was used to prepare poly-A–selected directional libraries using the Kapa Stranded RNAseq Library Preparation Kit (KAPABIOSYSTEMS), and libraries were sequenced utilizing the Illumina Nextseq platform at the Oncogenomics Sequencing Core of the University of Miami (Miami, FL).

Data were analyzed on the Illumina Basespace platform utilizing RNA express pipeline. Briefly, RNA-sequencing (RNA-seq) reads were aligned to the human genome (hg19) using STAR aligner, and subsequently aligned reads were assigned to genes, followed by differential gene expression analysis performed using DESeq2 between all conditions. Cufflinks package was used to calculate FPKM values for heat map representation.

We performed (i) gene-set analysis to identify significantly enriched gene sets for the differentially expressed genes (log_2_ fold change > 0.5; *P*_adj_ < 0.05) in a pair-wise fashion between all four experimental conditions; and (ii) gene-set enrichment analysis (GSEA) using the R package *fgsea*. Enrichment scores for more than 10,000 gene sets previously compiled from various sources were calculated for this analysis (Supplementary Table S1; ref. 3).

The expression data have been deposited in the NCBI's Gene Expression Omnibus and are accessible through GEO Series accession number (GSE138349).

### IHC and Immunofluorescence

Formalin-fixed paraffin-embedded (FFPE) EMZL tissues were used for IHC studies (29 OAMZL and 35 EMZL not originating in ocular adnexa). Tissue sections were cut from FFPE tissue blocks at 4-μm thickness and mounted on positively charged slides. IHC was performed using a standard protocol as previously described (53). Briefly, tissue sections underwent heat-induced epitope retrieval in pH 6.0 citrate buffer (Dako). Endogenous peroxidase was blocked by 3% H_2_O_2_ solution for 10 minutes. Staining was performed overnight using CABIN1 rabbit mAb (Cell Signaling Technology, #12660S) and NFATc1 (BD Biosciences, #556602) at 1:100 dilutions. Each case was analyzed using standard microscopy by one hematopathologist (blinded to molecular data). For the IHC assessment of CABIN1 expression in OAMZL and extranodal MZL in other anatomic sites, we analyzed the percentage of tumor cells positive for CABIN1 as 25% increments (score 1, 25%; 2, 26%–49%; 3, 50%–74%; and 4, ≥75%). To assess for differences in CABIN expression between OAMZL cases with and without CABIN1 mutations/deletions, we also determined the intensity of nuclear staining as mild to intense (+ vs. ++). Cases with score of 0 and 1 were defined as low expressors.

Immunofluorescence labeling was performed using FFPE tissue sections on which CABIN1 staining was performed in combination with the CD20 or CD3 labeling. Slides were incubated with rabbit anti-CABIN1 antibody and with mouse anti-CD20 (Dako) or mouse anti-CD3 (Dako) for 2 hours at room temperature. Alexa Fluor 594 (red) goat anti-rabbit IgG conjugate and Alexa Fluor 488 (green) goat antibody anti-mouse IgG conjugate antibodies (Life Technologies) were used as secondary antibodies. The nuclei were counterstained with 4′,6-diamidino-2-phenylindole. After aqueous mounting, the slides were observed using a fluorescence microscope (Olympus BX51).

Immunofluorescence staining of NFATc1 was performed as described previously (54). Briefly, tissue sections were deparaffinized, hydrated, and then underwent heat-induced epitope antigen retrieval in a steamer for 20 minutes. Tissue sections were permeabilized in PBS containing 1% Triton X-100 for 30 minutes, blocked with Image IT FX signal enhancer (Invitrogen), and incubated with mouse NFATc1 primary antibody (R&D Systems; 1:50) overnight at 4°C. Alexa Fluor 488 donkey anti-mouse IgG (Invitrogen), was applied at 1:1,000 in PBS for 45 minutes followed by three PBS washes for 5 minutes. Images were acquired using Vectra Polaris Automated Quantitative Pathology Imaging System in the Flow Cytometry & Cellular Imaging Core Facility (MD Anderson Cancer Center, Houston, TX). Image Analysis of the nuclear immunofluorescence intensities were performed with ImageJ software (NIH, Bethesda, MD). Immunofluorescence intensities were quantified in 50 to 60 cells in each specimen.

### Cells, Plasmids, and Lentivirus Production

The splenic MZL cell line SSK41 was a kind gift of Drs. Bertoni and Rossi from the Institute of Oncology Research (IOR), Bellinzona, Switzerland. Cells were grown in RPMI media (Thermo Fisher Scientific) supplemented with 10% FBS and 1% penicillin–streptomycin (10,000 U/mL). For CABIN1 KD experiments, two different short hairpin RNA (shRNA) targeting CABIN1 3′UTR (shRNA1 sense: 5′-CCGCCTTAGCCATGTGAAG-3′; antisense: 5′-CTTCACATGGCTAAGGCGG-3′; shRNA2 sense: 5′-CCAGAGGCCCACATGGATG-3′; antisense: 5′-CATCCATGTGGGCCTCTGG-3′) were designed and cloned into pLL3.7 vector (Addgene). Scrambled shRNA was used as a control. For CABIN1 (Uniprot B5MEB3) overexpression experiments, human influenza hemagglutinin (HA)-tagged CABIN1 was synthesized by GeneArt (Thermo Fisher Scientific), and the Q5 Site-Directed Mutagenesis Kit (New England Biolabs) was used to create 5 CABIN1 mutants (NM_001201429: c.C5041T, c.C5722T, c.G5750T, c.A6053G, c.C6486G). CABIN1 WT and mutants were then subcloned in the lentiviral expression vector pLVX-TetOne (Clontech) using standard techniques.

For the production of lentiviral particles, lentiviral vectors were transfected into HEK293 cells together with VSV-G envelope expressing (pMD2.G) and packaging (psPAX2) plasmids (Addgene). Virus-containing media were collected at 48 and 72 hours posttransfection and viruses were concentrated using Lenti-X concentrator (TakaraBio). Transduction of SSK41 cells was done in the presence of the cationic additive protamine sulfate (Sigma Aldrich). Cell selection was done by GFP sorting for cells transduced with pLL3.7 vector and puromycin selection (1 μg/mL) for cells transduced with pLVX-TetOne vector.

### Luciferase Reporter Assay

SSK41 cells were cotransfected with either the *NFAT* or *MEF2* promoter luciferase reporters (Addgene) and the internal control plasmid pRLTK (Promega) using Amaxa machine protocol M13 and Kit V (Lonza). Forty-eight hours after transfection, cells were placed in serum-free media and stimulated with 20 μg/mL of goat anti-human IgM F(ab’)_2_ Fc (α-IgM F(ab’)_2_) (Thermo Fisher Scientific, H15100). After 4 hours of stimulation, firefly and *Renilla* luciferase activities were measured with the Dual Luciferase Assay Kit (Promega).

### Growth Inhibition Assay

The splenic MZL cell line SSK41 (a kind gift of Drs. Bertoni and Rossi from the Institute of Oncology Research (IOR), Bellinzona Switzerland) or HEK293 cells (acquired from ATCC in 2010), were seeded on a white 96-well plate at concentrations of 10,000 cells/well, sufficient to have untreated cells in exponential growth during the experiment. Cells were treated for 48 to 96 hours with increasing concentrations of Cyclosporin A (Sigma Aldrich, #30024). Cell viability was determined using the CellTiter-Glo (Promega) measured with the SynergyHT Microplate Reader (BioTek). Cell apoptosis and death were determined by flow cytometry following labeling with propidium iodide and 7-amino-actinomycin D and cell-cycle analysis performed, as previously reported (53). Cell lines were regularly tested for *Mycoplasma* infection (MycoAlert Mycoplasma Detection Kit, Lonza) at 3-month intervals and genotyped by short tandem repeat DNA profiling annually and at completion of the project.

### Western Blotting and Immunoprecipitation

Cells were lysed in 2.5% SDS, 250 mmol/L Tris-HCl, pH 7.4, at 95°C. Gel electrophoresis and immunoblotting were done as previously described (53). Immunoblots were developed using primary antibodies directed to CABIN1 [Cell Signaling Technology (D2B9F), pan-Calcineurin A (Cell Signaling Technology, 2614S), calmodulin (Abcam, EP799Y), SIN3A (Abcam, ab3479), MEF2B (Abcam, EPR22193–26), and GAPDH (Santa Cruz Biotechnology, sc-32233, Inc)] and horseradish peroxidase–conjugated secondary antibodies. Immunoprecipitations were performed as previously reported (53) using indicated antibodies.

### Statistical Analysis

Statistical analyses were performed using ANOVA followed by Tukey *post hoc* test for comparing luciferase reporter activities, *t* test for comparing Western blot densitometry, nuclear NFATc1 immunofluorescence intensity, and by Fisher exact test for comparing CABIN1 and NFAT expression and to calculate the enrichment of the differentially expressed genes. Comparison of cell viability at a fixed concentration of cyclosporin A between cell types were performed by ANOVA followed by pairwise comparisons versus CABIN1shRNA as the reference using the Dunnett method for multiple comparisons. AUC of concentration–cell viability for each replicate were compared by ANOVA following pairwise comparisons versus CABIN1shRNA using the Dunnett methods for multiple comparisons. A two-sided *P* < 0.05 was considered statistically significant.

### Data Availability Statement

Data were generated by the authors, with processed data included in the article. Raw transcriptome sequencing data were deposited in the NCBI's Gene Expression Omnibus and are accessible through GEO Series accession number (GSE138349). Raw exome sequencing data were deposited in the European Genome-Phenome Archive under the dataset number (EGAD00001011067).

## Results

### Identification of Candidate Cancer Driver Genes in OAMZL

We utilized WES to identify somatic nonsilent mutations and CN alterations in 69 DNA samples of OAMZL and 7 germline DNA controls (6 paired normal and one additional normal sample from a patient whose tumor DNA did not pass quality control). We filtered out variants found with a frequency more than 1% in control populations, and selected variants that were exonic, not synonymous, and deleterious. To identify possible candidate cancer driver genes (CCDG), we focused on those genes carrying genetic mutations (SNPs and short insertions or deletions) in 10% or more of the analyzed tumor samples. Genes that are highly polymorphic and are likely to harbor false-positive variants in WES data were excluded from our driver gene analysis as previously reported (43, 44). We combined the mutation calls with significant focal CN calls and identified 667 variants (577 missense mutations and 90 truncating mutations) and 213 CN focal alterations (162 CN gains and 51 CN losses) across 74 putative driver genes (Fig. 1; Supplementary Fig. S2; Supplementary Tables S2 and S3). Genes with at least one variant identified through the analysis of samples with paired normal tissue are labeled as “altered in T-N paired sample”. Among the most commonly mutated genes, we detected genes previously reported to be altered in OAMZL studies using targeted sequencing [e.g., *TNFAIP3* (A20; 26%), *TBL1XR1* (22%), and *CREBBP* (17%); refs. 29, 31], confirming our filtering strategy. Previously described tumor suppressor genes like *TNFAIP3* (26%) show a typical pattern of alterations consisting of CN losses (6q23.3 deletion in 10%) and/or truncating mutations. The *TNFAIP3* mutations and CN losses observed in the analyzed specimens were previously detected in the same specimens by Sanger Sequencing and TaqMan Copy Number Assay (assay ID Hs06775497), respectively, as reported by us (26), validating our methodology and results. Moreover, we observed previously reported trisomy of chromosomes 3 and 18, with amplification of both p and q arms, in 2 (3%) and 3 (4.3%) samples, respectively (Supplementary Table S3). Furthermore, by applying this approach to a large collection DNA samples from fresh OAMZL tissues, the majority of the detected altered genes are novel and previously not reported, including mutations and deletion in Calcineurin binding protein 1 (*CABIN1*, 30%), and truncating mutations and CN gains in *RHOA* (26%). The latter included 2 specific focal gains of *RHOA*, 2 amplifications of the 3p arm, and 3 larger chromosome 3 gains. We did not observe *RHOA* G17V mutations, but one specimen harbored a stop gain at G17.

Combining DNA mutations and CN alterations, the number of genetic lesions per tumor sample in the 74 proposed driver genes ranged from 4 to 52, with an average load of 12.5 lesions per case, which is consistent with previous genetic studies in other types of MZL lymphoma (2, 27) but higher than previously reported in OAMZL using targeting sequencing (29–31).

**FIGURE 2:**
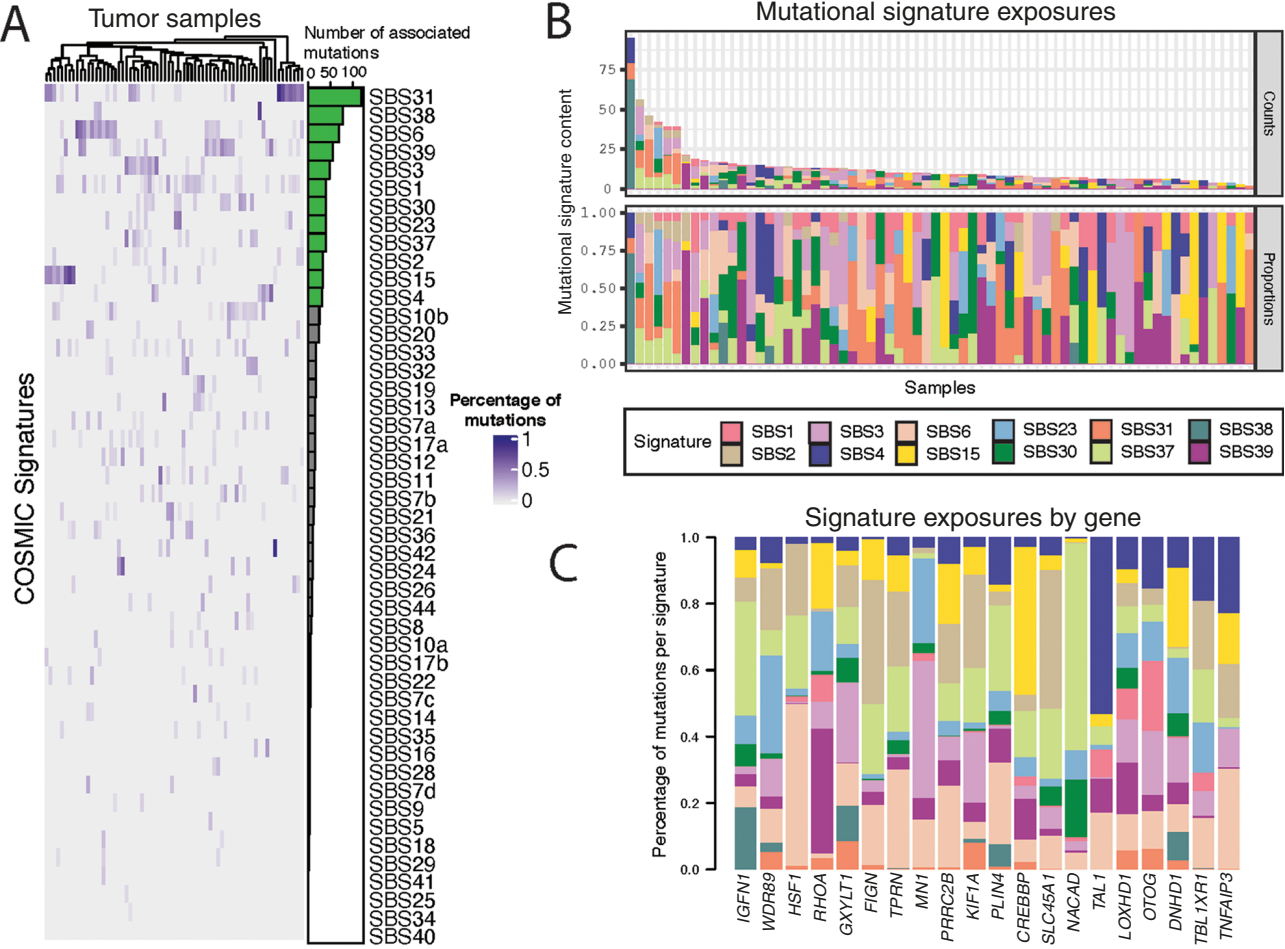
Characterization of mutational processes. **A,** Heat map showing the contribution of each of the 47 COSMIC mutation signatures to each individual tumor. **B,** Number (top) and proportion (bottom) of mutations in each tumor that are attributed to the top 12 most prevalent signatures. **C,** Relative enrichment of the top 12 most prevalent signatures in the 20 most recurrently mutated genes.

Multiple mutational signatures have been described in cancers as the result of different mutational processes (51). To evaluate the contribution of previously described mutational signatures (COSMIC) to each individual tumor genome, we used Palimpsest to estimate the exposures of these mutational signatures (Fig. 2A and B). This showed three samples that demonstrated mutation signatures associated with the indirect effect of ultraviolet light, with one of them showing a very high number of mutations corresponding to this signature. This specimen had a normal paired sample with a much higher than average number of Mutect variant calls, likely due to UV light exposure. These 3 samples were derived from patients residing in Florida. We also found enrichment for mutations that are associated with defective DNA mismatch repair (signatures 6 and 15) and mutations due to defective homologous recombination DNA damage repair (signature 3) in a subset of samples, suggesting that these cells are proliferating B cells and may have experienced a germinal center reaction (ref. 53; Fig. 2A and B).

Next, we explored the relative contribution of the 12 most prevalent signatures on the top 20 recurrently mutated genes (Fig. 2C). While these recurrently mutated genes showed predominance of mutations caused by failure of DNA double-strand break repair by homologous recombination (signature 3), many genes were enriched for mutations arising from different mutational processes.

### Alterations in MZ Development Pathways

**FIGURE 3:**
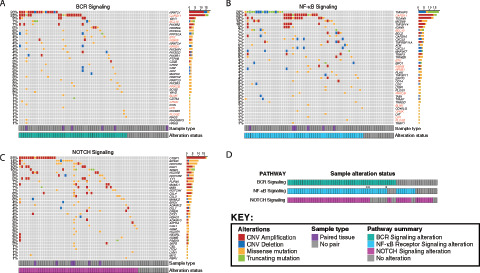
Oncoprints showing alterations occurring at any frequency in OAMZL tumors in genes belonging to MZ development pathways. **A,** BCR signaling. **B,** NF-κB signaling. Genes marked in red in **A** and **B** are found in both the BCR and NF-κB signaling pathways. **C,** NOTCH signaling. Columns correspond to individual patients while genes and their alteration frequency are listed in rows. **D,** Cooccurrence of genetic alterations of BCR, NF-κB, and NOTCH signaling pathways in individual tumors. Samples that are exclusively altered in the same set of genes shared by BCR and NF-κB signaling pathways are marked with asterisks. The color code for each type of alteration is illustrated in the figure key.

BCR, NF-κB, and NOTCH pathways are implicated in normal biology of MZ B cells (13). To comprehensively assess mutations in these pathways and their potential implications in the pathogenesis of OAMZL, we next focused on genes belonging to these pathways harboring mutations in at least one sample (Supplementary Tables S2 and S4). These mutations were merged with CN variant calls for the altered genes and are presented in Fig. 3. Some of the genes belonging to these pathways play roles in more than one pathway and alterations in these genes may deregulate more than one pathway.

We identified alterations in components of the BCR signaling in 72.5% of the cases that occurred in a mutually exclusive manner in 18 of 50 affected patients (36%; Fig. 3A). We identified mutations and novel CN gains of *CARD11* in 25% of cases. This gene is a component of the BCR signaling pathway that also functions as a positive regulator of the NF-κB pathway. While mutations of *CARD11* have been previously implicated in the pathogenesis of DLBCL, the effects of mutations and CN gains in OAMZL are unknown (55).

We confirmed previously reported alterations in several components of the NF-κB signaling, including *TNFAIP3A* (26%), *MYD88* (12%), and *BCL10* (7%), which were observed in 52 tumor specimens (Fig. 3B). Mutations in the NF-κB signaling genes tended to cooccur in 35 tumors (67.3%), ranging from 0 to 10 altered genes per patient, with an average of 2.1.

Like previous genetic studies in nodal and splenic MZL (2, 28), we discovered recurrent mutations and CN changes in the NOTCH signaling pathway (Fig. 3C). However, differently from previous studies, *CTBP1* (25%) was the most altered NOTCH gene in OAMZL followed by *EP300* (14%), *NOTCH3* (13%; Supplementary Fig. S3), *DLK1* (10%), and *SPEN* (10%). Mutations in NOTCH signaling genes cooccurred in most cases (*n* = 55, 80%), ranging from 0 to 16 altered genes per patient, with an average of 2.0 affected genes.

Interestingly, most tumors harbored simultaneous alterations in both BCR and NFκB (Fig. 3D). The latter can be activated downstream of BCR and by other pathways, indicating the need for multiple “hits” in these pathways for OAMZL pathogenesis. We noticed that many altered genes are shared across the BCR and NF-κB signaling pathways and have been marked in red in Fig. 3A and B. In addition, three samples harbored alterations in this set in the same genes exclusively. Because these genes are known to impact signaling in both pathways, we have counted these samples as altered in both pathways for the purposes of Fig. 3D, but have marked them with an asterisk. Furthermore, we observed concomitant alterations in the NOTCH signaling pathways, suggesting cooperative involvement in OAMZL pathogenesis. Overall, these observations show that pathways implicated in normal function of MZ cells may be mutated in all the subtypes of MZL, but specific genes are affected differently in the nodal and splenic lymphomas in comparison to OAMZL.

### CABIN1 Dysregulation and Expression in OAMZL

**FIGURE 4:**
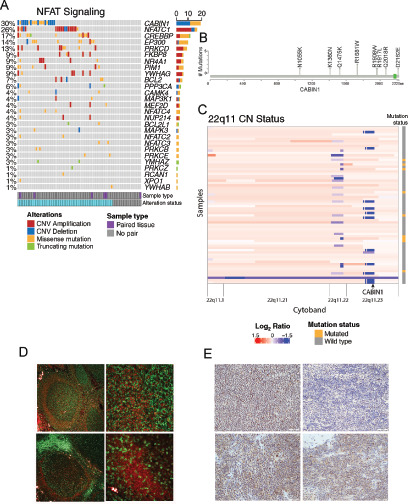
CABIN1 alterations and expression. **A,** Oncoprint showing alterations occurring across genes in the NFAT signaling pathway. Columns correspond to patients while genes are listed in rows. **B,** Lollipop plot showing the somatic nonsilent mutations found in *CABIN1* gene with annotations of the amino acid changes. **C,** Heat map of CN status for cytoband 22q11 including the CABIN1 locus (annotated). Across this cytoband, segment colors vary from blue for strong CN losses to red for strong CN gains. Each row represents a sample, and each column represents a reported CN segment. Sample mutation status is noted to the right. **D,** Immunofluorescence expression analysis of CABIN1 (red), CD20 (green, top), and CD3 (green, bottom) in normal lymph node. Magnification: top and bottom left, 4×; top and bottom right, 40×. **E,** IHC analysis of CABIN1 expression in different MALT lymphomas (magnification all 20×). Top left, OAMZL with WT CABIN1; top right, OAMZL with CABIN1 CN losses; bottom left, parotid MZL; bottom right, lung MZL. White bars, 50 μm.

We observed mostly mutually exclusive mutations in the four members of the NFAT family of transcription factors (Fig. 3A). These NFAT gene mutations, together with frequently observed mutations in the NFAT target genes (76.8% of the cases; Fig. 4A; Supplementary Table S4) suggest that deregulation of the NFAT signaling at multiple levels is a common novel alteration in OAMZL, while it is only rarely observed in other lymphomas. NFAT pathway mutations occurred in a mutually exclusive manner in 18 of 53 affected patients (34.0%).

*CABIN1* is one of the most commonly altered genes in OAMZL in general, specifically present in the NFAT pathway. Normally, CABIN1 binds to and inhibits Calcineurin-mediated signal transduction, thus functioning as a negative regulator of NFAT signaling (56, 57). In OAMZL samples, *CABIN1* was affected by missense mutations in 9 different tumor samples (Fig. 4B), two of which were configured as somatic events that occurred in samples with paired normal tissue. Two of these mutations (c.A6053G and c.C6486G, based on CABIN1 sequence NM_001201429) were located in PEST sequences annotated by Sun and colleagues (56). PEST sequences have been proposed to target a wide variety of cellular proteins for degradation but may have additional functions. In addition, we identified a *CABIN1* CN loss in 12 more nonoverlapping samples. This deletion is a focal loss that does not affect the nearby Immunoglobulin Lambda locus for 11 of 12 patients (Fig. 4C). Finally, while *CABIN1* was previously reported to be mutated in only 4% of DLBCL samples, an unbiased CRISPR screen of six lymphoma cell lines found it to be an essential gene with tumor suppressor behavior (3). These findings would suggest the importance of CABIN1 dysregulation in the oncogenesis of OAMZL. Therefore, we decided to focus on characterizing the biological effects of *CABIN1* mutation and CN loss in OAMZL. Immunofluorescence staining of normal lymph nodes revealed that CABIN1 is expressed in CD20^+^ B cells of the mantle zone of the germinal center (Fig. 4D, top), while it is not expressed in CD3^+^ T cells. Of note, in human lymph nodes, MZ is not well discernable from the mantle zone. IHC of normal spleen demonstrated variable dim expression in a subset of germinal center B cells, mantle zone B cells, and MZ B cells (Supplementary Fig. S4). Next, we used IHC to assess CABIN1 protein level in our cohort of OAMZL (29 specimens with available tissue) and other MALT lymphomas (35 samples). CABIN1 was expressed in larger number of cells and with higher intensity in 13 of 14 OAMZL cases with WT CABIN1, compared with 6 of 15 cases carrying gene deletions or mutations (*P* = 0.038; Fig. 4E, top; Supplementary Table S5). Among the low CABIN1 expressors, 5 harbored deletion and one mutation (G4080C). OAMZL cases expressed CABIN1 less frequently than MALT lymphomas originating in other anatomic locations, the latter uniformly expressing CABIN1 (*P* = 0.0009; Fig. 4E, bottom). Overall, our data show that CABIN1 is expressed in MALT lymphomas and that CABIN1 CN losses/mutations cause a decrease in the expression of CABIN1 in some OAMZL.

### CABIN1 Regulates NFAT and MEF2B Signaling Upon BCR Stimulation in MZL

**FIGURE 5:**
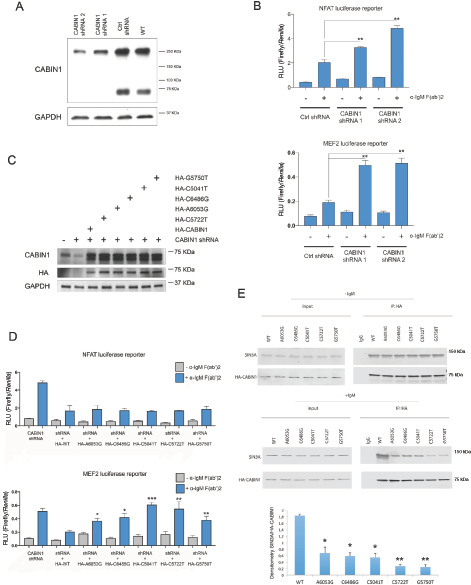
Effects of CABIN1 deletion and mutations on the NFAT and MEF2B transcriptional activities. **A,** Western blot analysis of CABIN1 expression in WT SSK41 cells or in SSK41 cells transduced with lentiviral vectors expressing either a control shRNA or CABIN1-specific shRNAs. The expression of the housekeeping gene *GAPDH* was used as a loading control. **B,** Luciferase reporter assay for NFAT (top) and MEF2 (bottom) transcriptional activity in SSK41 cells transduced with lentiviral vectors expressing either a control shRNA or CABIN1-specific shRNAs. Where indicated, cells were stimulated for 4 hours with α-IgM F(ab’)_2_. *, *P* = 0.001; **, *P* < 0.0002. **C,** Western blot analysis of CABIN1 expression in WT SSK41 cells or in SSK41 cells in which CABIN1 was initially knocked down using 3′-UTR targeting shRNA followed by expression of HA-tagged CABIN1 WT or mutants. CABIN1 was detected using anti-CABIN1 and anti-HA antibodies, while expression of the housekeeping gene *GAPDH* was used as a loading control. **D,** Luciferase reporter assay for NFAT (top) and MEF2 (bottom) transcriptional activity in SSK41 CABIN1 KD cells and cells expressing indicated CABIN1 constructs as shown in **C**. Where indicated, cells were stimulated for 4 hours with α-IgM F(ab’)_2_, in comparison to stimulated HA-WT: *, *P* < 0.005; **, *P* < 0.0001; ***, *P* = 0.00006. **E,** Representative immunoprecipitation (IP) assays with anti-HA antibodies using whole-cell protein extracts from unstimulated and α-IgM F(ab’)_2_–stimulated SSK141 cells expressing different CABIN1 mutants, as shown in **C** followed by Western blotting using indicated antibodies. Also shown mean and SD of relative SIN3A densitometry adjusted to immunoprecipitated CABIN1 in each cell type versus WT cells from three independent experiments. Statistical analyses of relative densitometry in mutants versus WT cells. *, *P* < 0.05; **, *P* < 0.01.

In normal, nonmalignant cells, CABIN1 has a dual role: (i) it directly binds to calcineurin and inhibits calcineurin-mediated activation of NFAT signal, (ii) it constitutively interacts with the transcription factor MEF2B to negatively regulate its transcriptional activity. These activities are mediated by the CABIN1 C-terminal domain (56, 58) that is present in the two isoforms expressed in the established splenic MZL cell lines and commonly mutated in OAMZL. MEF2B plays an important role in germinal center development and is reported to have an oncogenic function in DLBCL and FL where it is frequently mutated (59). We hypothesized that genetic inactivation of CABIN1 function by deletions or mutations may contribute to OAMZL pathogenesis by boosting BCR-mediated NFAT and/or MEF2 signaling. To understand the function of CABIN1 in MZL cells, we used shRNAs targeting its’ 3′ UTR to KD both isoforms of CABIN1 (Fig. 5A). We then used luciferase reporter systems to measure the transcriptional activities of NFAT and MEF2 in response to BCR stimulation. Supporting our hypothesis, α-IgM F(ab’)_2_ stimulation of CABIN1-depleted cells induced a more prominent activation of NFAT and MEF2 signaling compared with control cells (Fig. 5B).

We next examined the effects on NFAT and MEF2 signaling of five different mutations in the C-terminal domain of CABIN1 that were detected in primary OAMZL tumors (Fig. 4B). To this end, we HA-tagged and cloned the low molecular weight isoform of CABIN1 (Uniprot B5MEB3) into the lentiviral expression vector pLVX-TetOne and modified it by site-directed mutagenesis to generate the five different C-terminal mutations found in the OAMZL tumors. By transducing the SSK41 CABIN1 KD cells with lentiviruses expressing distinct CABIN1 mutants, we generated six stable cell lines that, in the presence of doxycycline, express either HA-CABIN1 WT or one of the five mutants (Fig. 5C). The presence of mutations in CABIN1 C-terminal domain did not decrease protein stability by Western blotting (Fig. 5C) with only mutant G5750T demonstrating lower stability in the cycloheximide chase assay (Supplementary Fig. S5). The A6053G mutant affecting PEST domain exhibited increased protein stability in cycloheximide chase assay compared with WT protein and had CABIN1 expression in the tumor like OAMZL tumors with WT CABIN1 based on IHC (Supplementary Table S5). These cell lines were used to analyze NFAT and MEF2 transcriptional activity with and without α-IgM F(ab’)_2_ stimulation by luciferase reporter systems.

Overexpression of CABIN1 WT significantly reduced NFAT reporter activity in response to α-IgM F(ab’)_2_ stimulation in comparison to CABIN1 KD cells (Fig. 5D, top). All the five mutants behaved as WT CABIN1 in reducing NFAT activity, thus suggesting that these mutations do not affect this signaling pathway (Fig. 5D, top). Furthermore, we did not observe differences in coimmunoprecipitation between calcineurin and WT and mutant CABIN1 proteins (Supplementary Fig. S6), thus explaining similar NFAT reporter activities in the presence of CABIN1 mutants.

Overexpression of CABIN1 WT also significantly reduced MEF2B activity in response to α-IgM F(ab’)_2_ stimulation when compared with CABIN1 KD cells (Fig. 5D, bottom). However, overexpression of the five mutants in CABIN1 KD cells failed to reduce α-IgM F(ab’)_2_-stimulated MEF2 reporter activity to the same extent as the WT CABIN1 (Fig. 5D, bottom) and there was a statistically significant increase in α-IgM F(ab’)_2_–stimulated MEF2 reporter activity between CABIN1 KD cells that are reexpressing all the analyzed CABIN1 mutants compared to CABIN1 WT (Fig. 5D, bottom). Furthermore, there was no statistical difference in α-IgM F(ab’)_2_ stimulated MEF2 reporter activity between CABIN1 KD cells and cells reconstituted with C5041T and C5722T CABIN1 mutants, suggesting loss of function similar to deletion (Fig. 5D, bottom). The differences in the effects of mutants on the MEF2B and NFAT reporters might stem from different affinity of the mutants to the calmodulin which by binding to CABIN1 releases MEF2B allowing transcriptional activation, while directly binding and activating calcineurin irrespective of its binding to the CABIN1. However, coimmunoprecipitation experiments demonstrated similar interactions between calmodulin and CABIN1 mutants or WT CABIN1 upon α-IgM F(ab’)_2_ stimulation (Supplementary Fig. S6). Alternatively, as CABIN1-induced inhibition of MEF2B is mediated by formation of complex with the transcriptional repressor SIN3A, the observed increased MEF2B reporter activity upon α-IgM F(ab’)_2_ stimulation might stem from decreased interaction between CABIN1 mutants and SIN3A (60). Indeed, coimmunoprecipitation experiments demonstrated decreased binding of CABIN1 mutants to SIN3A in comparison to the WT CABIN 1 (Fig. 5E), thus explaining enhanced MEF2B reporter activity in cells expressing mutants in comparison to the CABIN1 WT (Fig. 5D, bottom).

To examine global gene expression changes that may be caused by genetic inactivation of CABIN1, we performed a transcriptomic analysis utilizing RNA-seq. RNA was extracted from SSK41 WT cells and SSK41 CABIN1 KD cells, before and after α-IgM F(ab’)_2_ stimulation. Expression analysis between WT cells versus CABIN1 KD cells showed 217 differentially expressed genes (log_2_ fold change > 0.5 and FDR < 0.01). Ninety-two of these genes were upregulated in CABIN1 KD cells and 125 were downregulated in comparison to WT cells (Supplementary Table S6). GO enrichment analysis showed enrichment for immune response (FDR = 2.85e^−11^) and BCR activity (FDR: 1.95e^−10^) among the most significantly enriched biological processes. GSEA showed that genes upregulated in KD cells are enriched for positive regulators of STAT cascade (*P*_adj_ = 0.047; NES = 1.73), while downregulated genes are enriched in antigen processing and presentation (*P*_adj_ = 0.0065; NES = −2.45), and in IFNα response (*P*_adj_ = 0.0065; NES, −2.56; Supplementary Fig. S7A).

**FIGURE 6:**
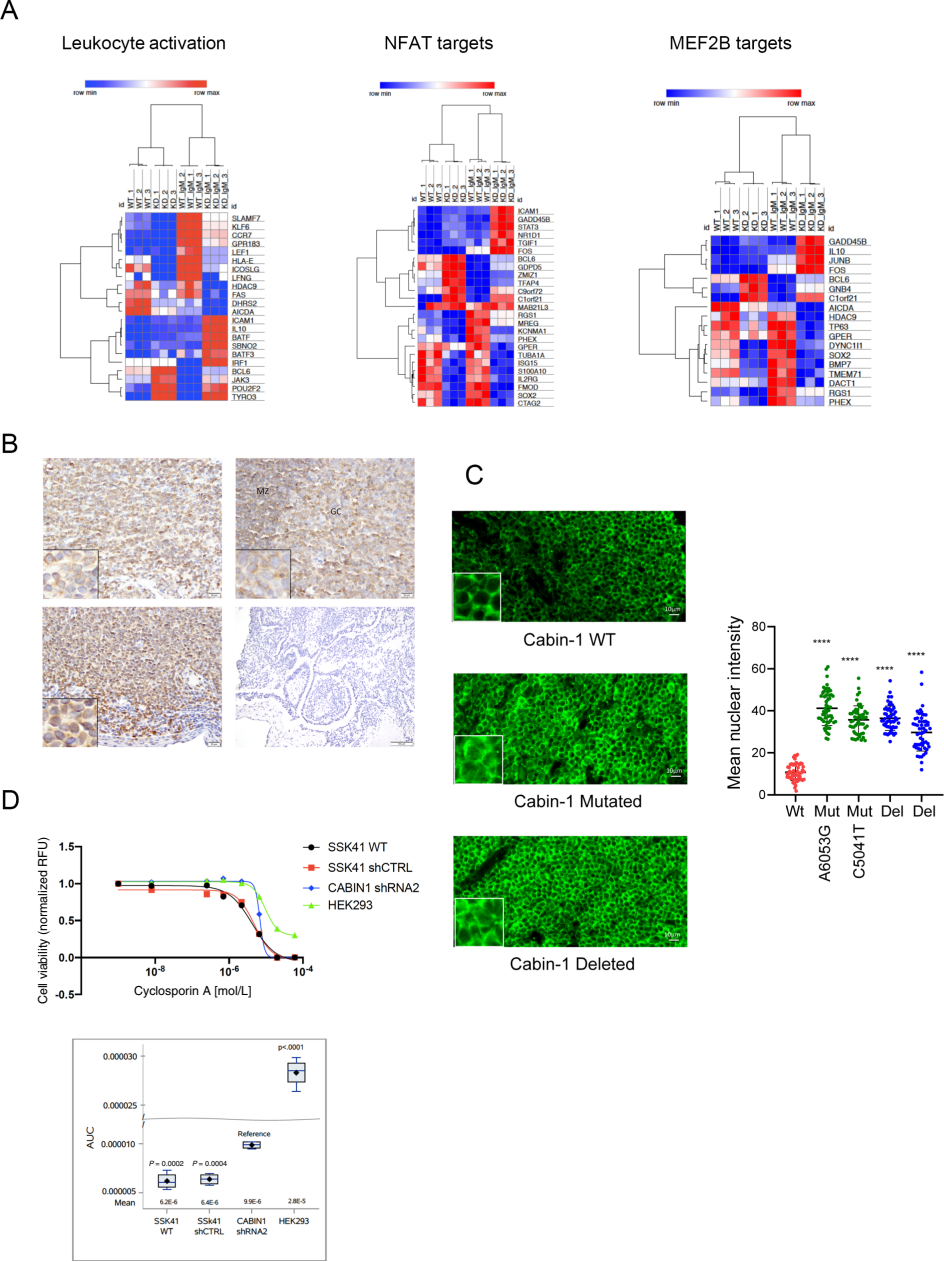
Effects of CABIN1 alterations on gene expression and NFAT activation. **A,** Heat maps showing FPKM values of genes that are differentially expressed in WT versus CABIN1 KD SSK41 cells after α-IgM F(ab’)_2_ stimulation. These genes are significantly enriched for leukocyte activation signature, NFAT and MAF2B targets. **B,** IHC analysis of NFAT expression and localization in OAMZL tumors with WT CABIN 1 (top left) and CABIN1 CN losses (bottom left). For control, NFAT antibody staining showed cytosolic expression in germinal center of normal lymph nodes (top right), while no staining detectable in the uterus (bottom right). Magnification: top left and right and bottom left, 40×; bottom right, 10×. Insets, 100×. **C,** NFATc1 (green) immunofluorescence in OAMZL tissue sections with WT and mutated CABIN1. Image analysis of mean nuclear fluorescence signal intensities were done using ImageJ software. ****, *P* < 0.0001. **D,** Cell viability assay of SSK41 WT, shRNA control, and CABIN1 KD cells treated for 72 hours with increasing concentrations of cyclosporin A. HEK293 cells are not sensitive to cyclosporin A and were used as control.

Expression analysis between α-IgM F(ab’)_2_–stimulated WT cells versus α-IgM F(ab’)_2_–stimulated CABIN1 KD cells identified 177 genes with differential expression (log_2_ fold change > 0.5 and FDR < 0.01). Eighty-seven of these genes were upregulated in the α-IgM F(ab’)_2_–stimulated CABIN1 KD cells and 90 were downregulated in comparison to the α-IgM F(ab’)_2_–stimulated CABIN1 WT cells (Supplementary Table S6). To attain a better picture of the transcriptional program activated by the α-IgM F(ab’)_2_ stimulation in CABIN1 KD cells, we examined the 177 differentially expressed genes using GSEA. Leukocyte activation was one of the most statistically enriched biological process (FDR = 2.8e^−17^; Fig. 6A) and among the upregulated genes belonging to this process we found IL10 and ICAM1, which are involved in promoting B-cell survival and proliferation through the regulation of the JAK/STAT signaling pathway and lowering the threshold of B-cell activation through the interaction with LFA1, respectively (61, 62). Differentially expressed genes were also enriched for NFAT (FDR = 3.04e^−7^) and MEF2B (FDR = 1.13e^−17^) targets, thus confirming our luciferase reporter analysis. GSEA showed that genes upregulated in α-IgM F(ab’)_2_-stimulated CABIN1 KD cells are enriched in the IL6, JAK, and STAT3 gene set (*P*_adj_ = 0.030; NES, 1.76), while downregulated genes are enriched in antigen processing and presentation (*P*_adj_ = 0.011; NES: −2.17), and in IFNα response (*P*_adj_ = 0.011; NES, −2.37; Supplementary Fig. S7B). All significantly enriched pathways (*P*_adj_ > 0.05) for pairwise GSEA across all four conditions (WT vs. KD, IgM WT vs. IgM KD, WT vs. IgM WT, KD vs. IgM KD) are reported in Supplementary Table S7.

These data suggest that genetic inactivation of CABIN1 causes an increased transcriptional activity of NFAT and MEF2, that in turn may result in a more pronounced activation of B cells through the enhanced activation of the IL6, JAK/STAT signaling pathway.

### CABIN1 CN Losses Cause Increased Activation of NFAT

Calcineurin activates NFAT signaling by dephosphorylating cytosolic NFAT and thus causing its translocation to the nucleus. Because CABIN1 inhibits calcineurin activity, we expected that CABIN1 CN loss in OAMZL might cause an increase in NFATc1 nuclear translocation. To test this hypothesis, we examined NFATc1 subcellular localization in OAMZL primary tumors using IHC. Supporting our hypothesis, all 8 tested OAMZL specimens with CABIN1 deletion and mutations showed presence of NFATc1 in the nucleus, while it was nuclear in only 2 of the 5 OAMZL specimens with CABIN1 WT (Fig. 6B; *P* = 0.012). Concordantly, IF studies confirmed increased nuclear NFATc1 in cases with deleted and mutated CABIN1 (Fig. 6C).

Because NFAT activation is mediated by the protein phosphatase calcineurin, we decided to examine activity of the calcineurin inhibitor cyclosporin A on MZL cell proliferation and apoptosis. In WT and CABIN1-deleted SSK41 MZL cells, cyclosporin A decreased cell viability to a greater extent than in nonlymphoma cells (HEK293; Fig. 6D), similar to recent reports in the ABC-Activated B cell DLBCL cell lines (63). Concordantly, cyclosporin A induced a dose-dependent apoptosis in both SSK41 cells transduced with control and CABIN1 shRNA, with effects being more pronounced in the SSK41 CABIN1 control shRNA cell, as we would expect, but without effect on cell cycle (Supplementary Fig. S8). These observations suggest that cyclosporin A can induce apoptosis in both CABIN1 WT and KD cells and should be tested for treatment of MZL tumors with NFAT and MEF2 activation.

## Discussion

The pathogenesis of EMZL at many anatomic locations, including the OAMZL, is still largely unknown. Determination of the tumor mutational landscape usually provides insights into pathogenesis. Herein we report the mutational landscape of the OAMZL established by WES of 69 *de novo* untreated fresh tumors. Our findings confirm involvement of the BCR and NF-κB signaling pathways in the pathogenesis of these tumors while identifying alterations in novel genes in these pathways. Furthermore, we unveil aberrations in new signaling pathways and cellular functions that likely contribute to OAMZL pathogenesis. These include aberrations in genes implicated in NOTCH signaling, and dysregulation of the calcium-induced NFAT and MEF2B activations, among others. NOTCH pathway is implicated in normal biology of MZ B cells (64) and recurrent mutations of *NOTCH2*, *NOTCH1*, and other genes in this pathway were previously reported in splenic and nodal MZL (2, 27, 28). However, while *NOTCH2* mutations were also detected in the OAMZL, *NOTCH3* (13%) was more commonly mutated in OAMZL. In contrast to previously reported *NOTCH2* and *NOTCH1* mutations that cluster near the C–terminal PEST-rich domain resulting in protein truncation or nonsynonymous substitutions affecting the extracellular heterodimerization domain (27, 28), *NOTCH3* mutations detected in the OAMZL are nonsynonymous substitutions affecting mainly extracellular heterodimerization domain (Supplementary Fig. S3). The function of these mutations needs to be evaluated in the future studies.

Mutations in the NFAT genes, their targets, and genes involved in the calcium-induced activation of the NFAT and MEF2B pathways are novel and previously not reported aberrations in MZ and other lymphomas. While *MEF2B* was reported to be mutated in DLBCL (59), the mechanism of activation revealed in OAMZL is novel. The NFAT pathway is activated upon stimulation of BCR and other signals increasing intracellular calcium. NFAT pathway regulates expression of genes encoding cytokines, components of signal transduction pathways, and transcriptional regulators that are critical for control of processes deregulated in cancer, such as proliferation, growth differentiation, and survival (65). Indeed, NFATc2-deficient mice are more susceptible to carcinogen-induced tumorigenesis (66). In DLBCL, NFATc1 may be constitutively activated (63, 67), and together with NF-κB cooperatively regulate expression of genes promoting cell proliferation and survival (68, 69). However, mutations in the NFAT genes were previously not reported in lymphomas. We observed missense mutations in *NFATc1*, *NFATc2*, *NFATc3*, and *NFATc4* genes in 7 (10%) OAMZL tumors. The functional effects of these mutations should be investigated in future studies.

In this study, we focused on another gene in this pathway, *CABIN1*, one of the most commonly mutated/deleted genes (32%) in OAMZL. CABIN1 is ubiquitously expressed, and its major functions are inhibition of calcineurin phosphatase activity in the cytoplasm and MEF2B transcriptional activity in the nucleus (56–58, 60, 70, 71). In nonstimulated cells, calcineurin is inhibited by its autoinhibitory domain. Upon calcium signaling, activated calmodulin binds to calcineurin via calmodulin binding domain adjacent to the autoinhibitory domain, causing dissociation of the autoinhibitory domain from the active site of calcineurin and its activation toward cellular substrates (e.g., NFAT; ref. 57). CABIN1 via its minimal calcineurin-binding domain (amino acids 2144–2157) contributes to the inhibition of calcineurin phosphatase activity (56). This inhibition is not mutually exclusive with effects of calmodulin on calcineurin and is not affected by CABIN1 binding to the calmodulin. Concordantly, overexpression of the C-terminal part of CABIN1 containing the calcineurin-binding domain is sufficient to block T-cell receptor–mediated IL2 promoter–driven luciferase reporter gene (56). However, transgenic animals expressing C-terminally truncated CABIN1 mutant not able to bind to calcineurin exhibited no gross changes in calcineurin activity in comparison to WT littermates. However, this truncation affected MEF2D induction (70). Therefore, the precise mechanism by which CABIN1 inhibits calcineurin activity is not fully elucidated. In our experiments, we confirmed that deletion of CABIN1 enhances BCR-induced NFAT reporter activity, gene expression, and is associated with constitutive presence of NFATc1 in the nucleus of the primary OAMZL tumor cells. No difference was observed between the CABIN1 mutants and WT protein in reporter assays and coimmunoprecipitation with calcineurin, suggesting that the mutants may not affect NFAT signaling pathway. However, by IHC and immunofluorescence staining, we did observe increased nuclear presence of NFATc1 in some of the tumors with CABIN1 mutations.

**FIGURE 7:**
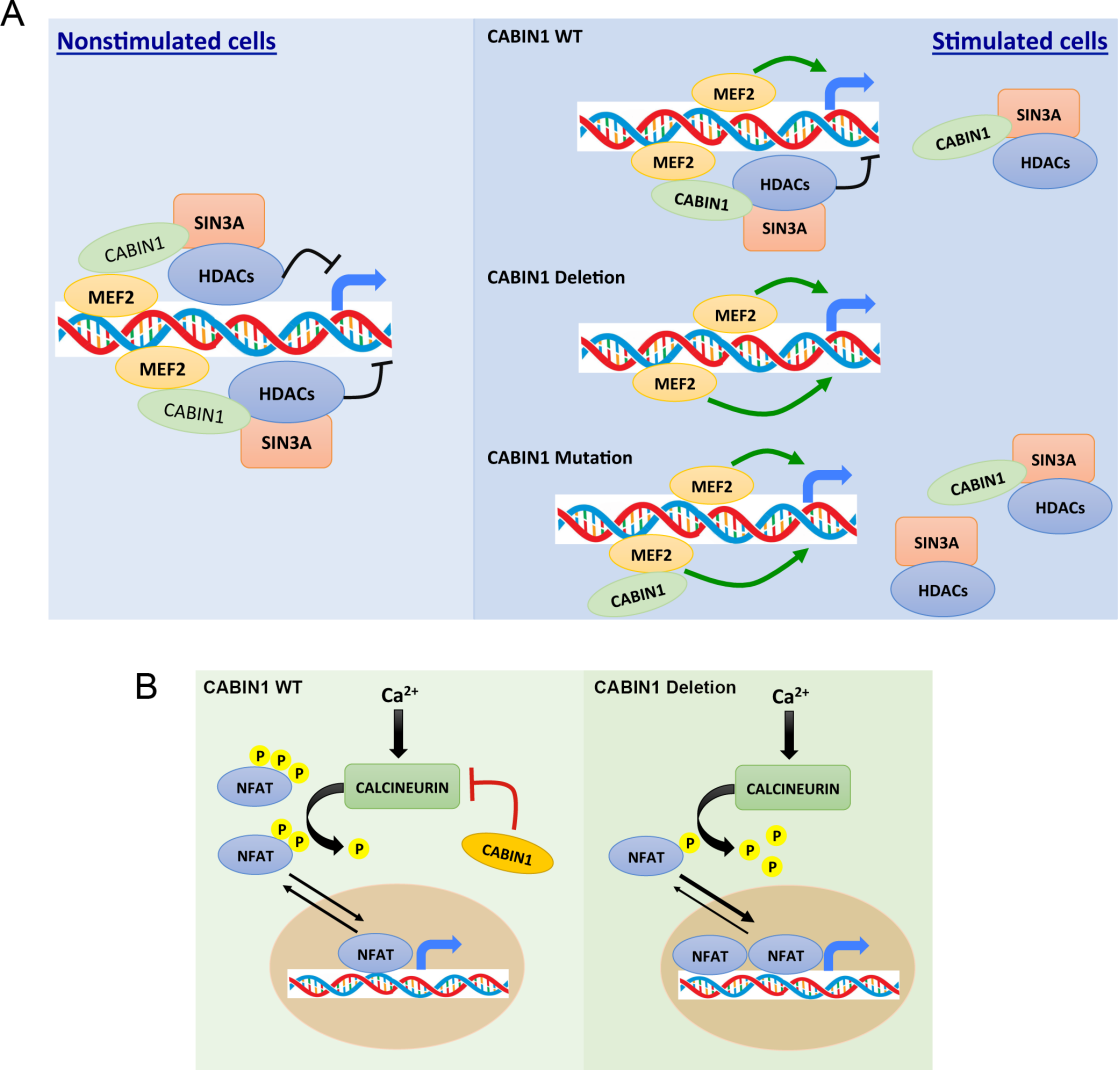
Schematic summary of effects of CABIN1 mutations and deletion on MEF2B (**A**) and NFAR (**B**) induced gene expression.

In contrast, the mechanism of CABIN1-mediated inhibition of the MEF2B transcriptional activity is different. MEF2B is constitutively bound to DNA regardless of the cell activation status (57, 71). In the absence of calcium signaling, MEF2B is associated with CABIN1 (amino acids 2157–2220) along with SIN3A/HDAC corepressor complex that silence the promoter (ref. 60; Fig. 7). Upon an increase in intracellular calcium concentration following BCR stimulation, the nuclear subset of calmodulin binds to CABIN1, releasing it together with SIN3A/HDAC corepressor complex from the MEF2B, allowing association of transcriptional coactivator p300 and inducing transcriptional activity (60). Consequently, CABIN1 interacts with MEF2B and calmodulin in a mutually exclusive manner and requires SIN3A/HDAC complex for MEF2B repression (60). Therefore, increased calmodulin binding to CABIN1 or decreased CABIN1 binding to SIN3A will alleviate its inhibitory effects and increase MEF2B transcriptional activity, but not calcineurin phosphatase activity. Concordantly, in our studies, we observed that deletion of CABIN1 enhances BCR-induced MEF2B reporter activity and gene expression. Furthermore, CABIN1 mutants by virtue of decreased affinity to SIN3A, as established in coimmunoprecipitation assay, also enhanced MEF2B reporter activity, like CABIN1 deletion. Overall, we demonstrate that by selecting specific mechanism of CABIN1 aberration deletion or mutations, OAMZL tumors may acquire simultaneous activation of both NFAT and MEF2B or only MEF2B. The biological and clinical significances of this difference is currently unknown and in the absence of *in vitro* and animal models of OAMZL is difficult to establish. However, we demonstrate that cyclosporin A induces apoptosis in CABIN1 WT and KD MZL cells, thus suggesting that it can be used as a novel therapeutic approach for MZL tumors with NFAT and MEF2 activation.

In summary, we established a mutational landscape of OAMZL confirming previous observations and revealing multiple novel genes and pathways that need to be studied to further demonstrate their role in pathogenesis of these tumors. Our findings also show some commonality in aberrations with previously reported genetic changes in splenic and nodal MZL, but also unveiled many unique changes for OAMZL. Further studies are needed to examine whether these novel mutations and CN changes are unique for OAMZL or are common to EMZL originating in other locations as well.

## Supplementary Material

Supplementary Figures Supplementary Figures 1-8

Supplementary Table 1 Sequencing Quality control (A), Mutational signature (B) and Geneset Sources (C)

Supplementary Table 2 Driver Gene mutations

Supplementary Table 3 Copy Number Variants Calling Results.

Supplementary Table 4 Pathway Variants Mutations shown in Figures 3 and 4.

Supplementary Table 5 CABIN1 expression in Mutants.

Supplementary Table 6 RNA expression in analyzed specimens.

Supplementary Table 7 GSEA results filter.
