# Correction: The Genetic Landscape of Ocular Adnexa MALT Lymphoma Reveals Frequent Aberrations in NFAT and MEF2B Signaling Pathways

**DOI:** 10.1158/2767-9764.CRC-23-0371

**Published:** 2023-08-29

**Authors:** Marco Magistri, Lanie E. Happ, Jeremy Ramdial, XiaoQing Lu, Vasileios Stathias, Kranthi Kunkalla, Nitin Agarwal, Xiaoyu Jiang, Stephan C. Schürer, Sander R. Dubovy, Jennifer R. Chapman, Francisco Vega, Sandeep Dave, Izidore S. Lossos

In the original version of this article (1), The accession number for the exome sequencing data was omitted from the data availability statement. The data are available in EGA under accession number EGAD00001011067. The error has been corrected in the latest online HTML and PDF versions of the article. The authors regret this error.

## References

[1] Magistri M , Happ LE, Ramdial J, Lu X, Stathias V, Kunkalla K, . The genetic landscape of ocular adnexa MALT lymphoma reveals frequent aberrations in NFAT and MEF2B signaling pathways. Cancer Res Commun 2021;1:1–16. 35528192 10.1158/2767-9764.CRC-21-0022 PMC9075502

