# Editor’s Note: The Genetic Landscape of Ocular Adnexa MALT Lymphoma Reveals Frequent Aberrations in NFAT and MEF2B Signaling Pathways

**DOI:** 10.1158/2767-9764.CRC-26-0217

**Published:** 2026-03-25

**Authors:** Marco Magistri, Lanie E. Happ, Jeremy Ramdial, XiaoQing Lu, Vasileios Stathias, Kranthi Kunkalla, Nitin Agarwal, Xiaoyu Jiang, Stephan C. Schürer, Sander R. Dubovy, Jennifer R. Chapman, Francisco Vega, Sandeep Dave, Izidore S. Lossos

The editors are publishing this note to alert readers to an issue about this article (1). This article states that two population frequency cutoff rates were used for analysis: <0.1% in the Materials and Methods section, and ≤1% in the Results section.

The authors clarified that the frequency cutoff rate stated in the Materials and Methods section is a typographical error and should be ≤1%, matching the frequency stated in the Results section. All analyses and results for potential driver events were subjected to a consistent population frequency cut-off of ≤1%.

Additionally, the control populations utilized for population frequency filtering were not explicitly described in the article. They are embedded in the Annovar tool that was used for variant annotation and cited in the article. The population database annotations from Annovar included ExAC for population filtering.
