# Supplementary material for: The Genetic Landscape of Ocular Adnexa MALT Lymphoma Reveals Frequent Aberrations in NFAT and MEF2B Signaling Pathways
Source: Cancer Res Commun. 2021 Oct 13;1(1):1–16. doi: 10.1158/2767-9764.CRC-21-0022 (PMC9075502; doi:10.1158/2767-9764.CRC-21-0022)
Supplement: Supplementary Figures — 1-8 [file crc-21-0022-s01.docx]

**Supplemental information**

Supplemental Information accompanies this paper of **Magistri et al**

**SUPPLEMENTAL TABLES**

Supplemental Table 1A-C: 7A) OAMZL sequencing quality control; 7B) Mutational signatures used in signature analysis; 7C) Sources for gene sets used in GSEA

Supplemental Table 2: Driver gene mutations

Supplemental Table 3: Copy Number variants

Supplemental Table 4: Pathway variants mutations

Supplemental Table 5: CABIN1 expression by immunohistochemistry in OAMZL tumors harboring CABIN1 deletions and mutations

Supplemental Table 6: Transcriptomic analysis utilizing RNA sequencing (RNAseq) of SSK41 WT and CABIN1 KD cells

Supplemental Table 7: All significantly enriched pathways (p-adj > 0.05) from GSEA between 4 experimental conditions: 1) WT vs KD; 2) IgM WT vs. IgM KD; 3) WT vs. IgM WT; 4) KD vs. IgM KD

**Supplemental Figures**


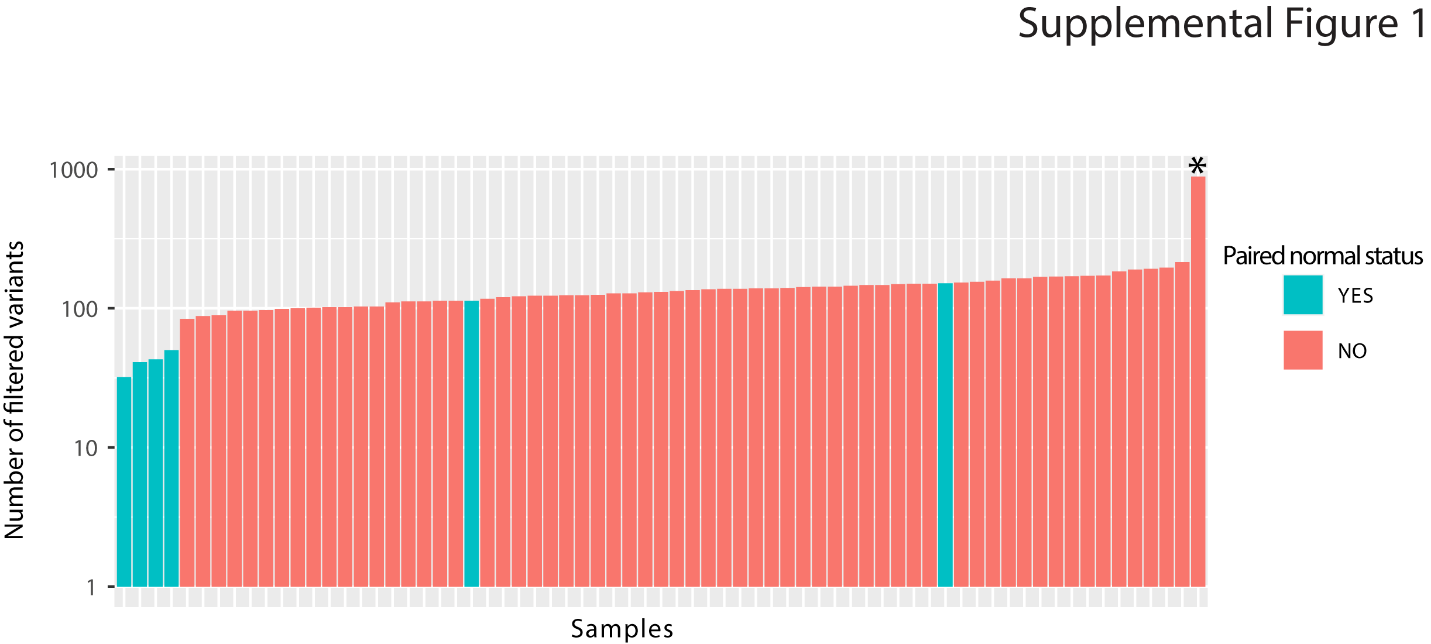


**Supplemental Figure 1. Number of HaplotypeCaller variants per sample after variant filtering.**

**
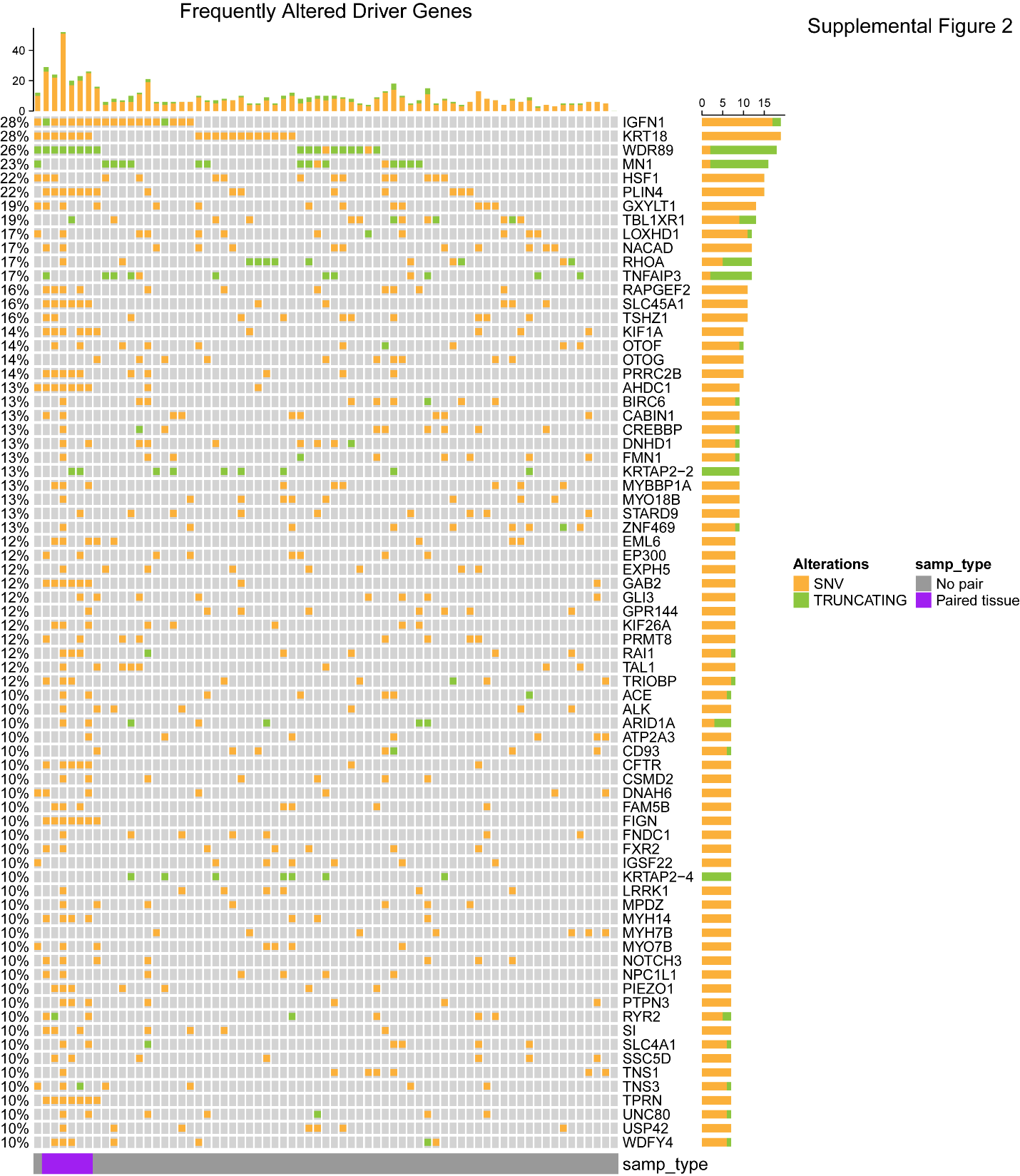
**

**Supplemental Figure 2.** An oncoprint showing the mutation status for the 74 most recurrently altered genes in 69 OAMZL tumors with frequency >= 10%. Each alteration type is color coded, as indicated in the figure. Each column corresponds to one sample and includes annotations at the bottom identifying those samples with paired normal tissue. Genes are represented in the rows.

**
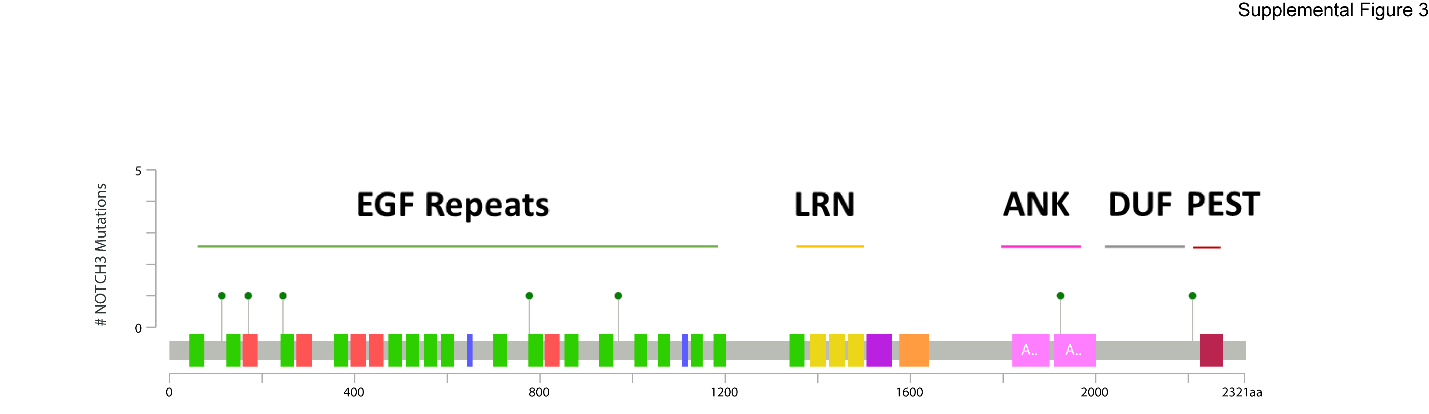
**

**Supplemental Figure 3. Lollipop plot showing the somatic non-silent mutations found in the NOTCH3 gene.** EGF- epidermal growth factor; LRN-cysteine-rich Lin repeats; ANK-ankyrin repeats; PEST- transactivation domain.

Supplemental Figure 4


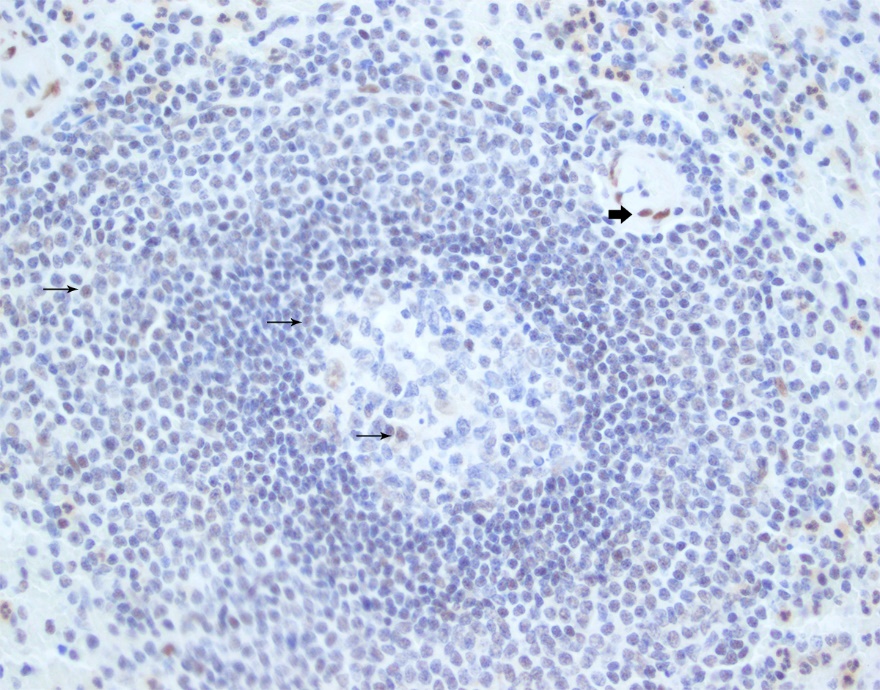


**Supplemental Figure 4. CABIN1 expression in normal spleen.** Immunohistochemistry for CABIN1 protein performed in normal spleen shows strong nuclear expression in normal endothelial cells (wide arrow) and variable dim expression in a subset of germinal center B-cells mantle zone B-cells and marginal zone B-cells (narrow arrows). Image is CABIN1 immunohistochemistry, 40x magnification.


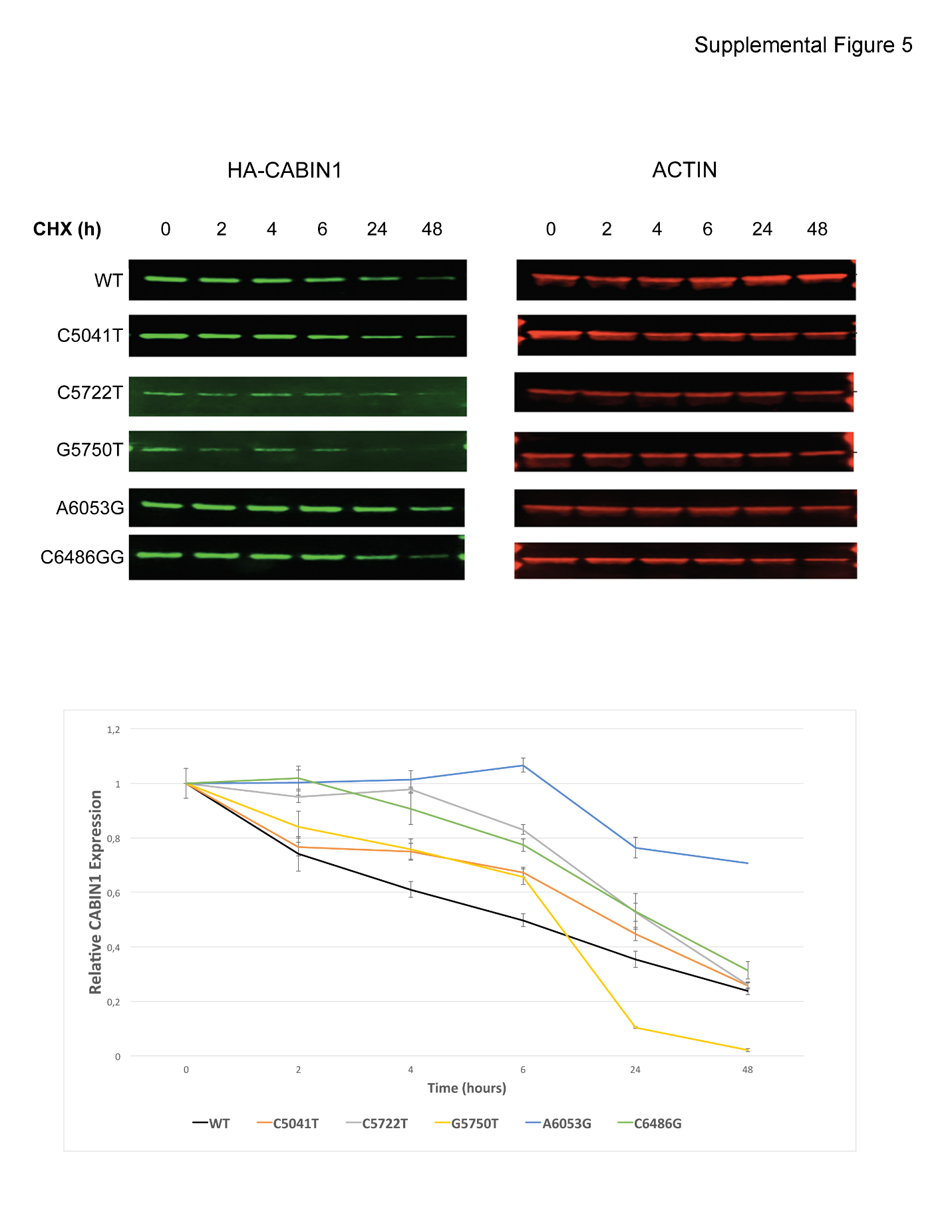


**Supplemental Figure 5. Cycloheximide protein chase assay for WT and mutants CABIN1 proteins.**

**
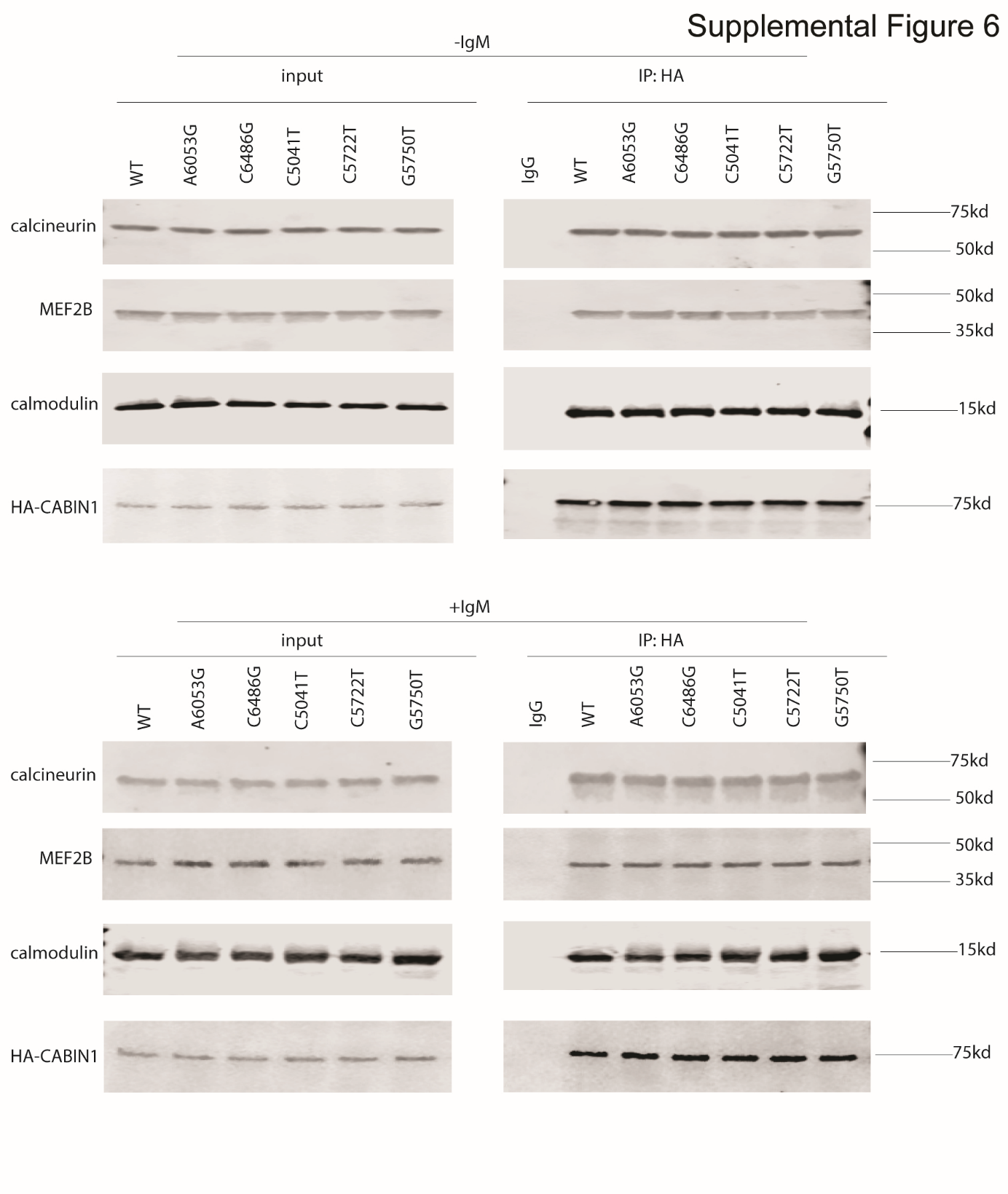
**

**Supplemental Figure 6. Interaction of CABIN1 mutants with the indicated proteins.**

Representative immunoprecipitation (IP) assays with anti-HA antibodies using whole cell protein extracts from resting and α-IgM F(ab’)_2_ stimulated SSK141 cells expressing different CABIN1 mutants, as shown in Figure 5, followed by western blotting using indicated antibodies.

Supplemental Figure 7


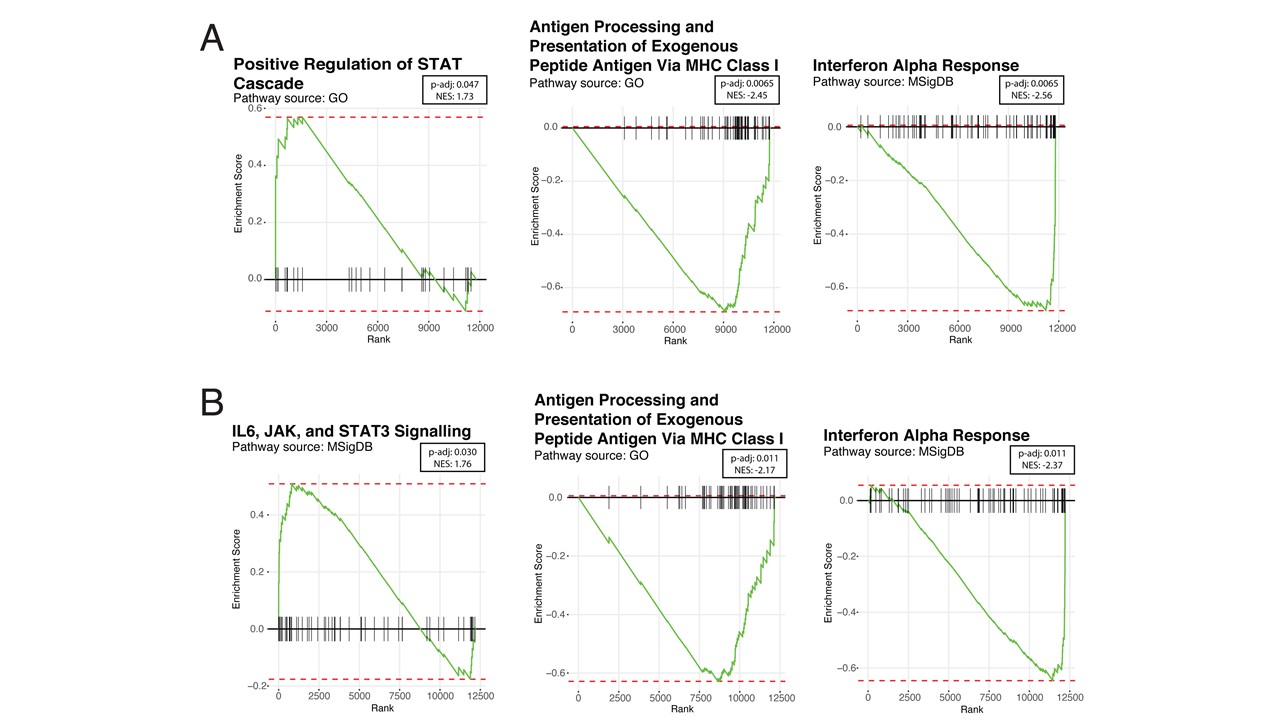


**Supplemental Figure 7. Enrichment plots for top gene sets following GSEA**

A) Enrichment plots of select enriched gene sets for SSK41 WT versus CABIN1 KD cells. All measured gene are ranked by their delta expression values between the two experimental conditions. The enrichment score (ES), representing the degree of over-representation of a gene set at the top or bottom of the ranked gene list, is shown on the y-axis and genes from the gene set are annotated as vertical black lines along the x-axis. The dotted red lines mark the maximum and minimum ES across the geneset. B) Enrichment plots of select enriched gene sets for IgM stimulated SSK41 WT versus CABIN1 KD cells.


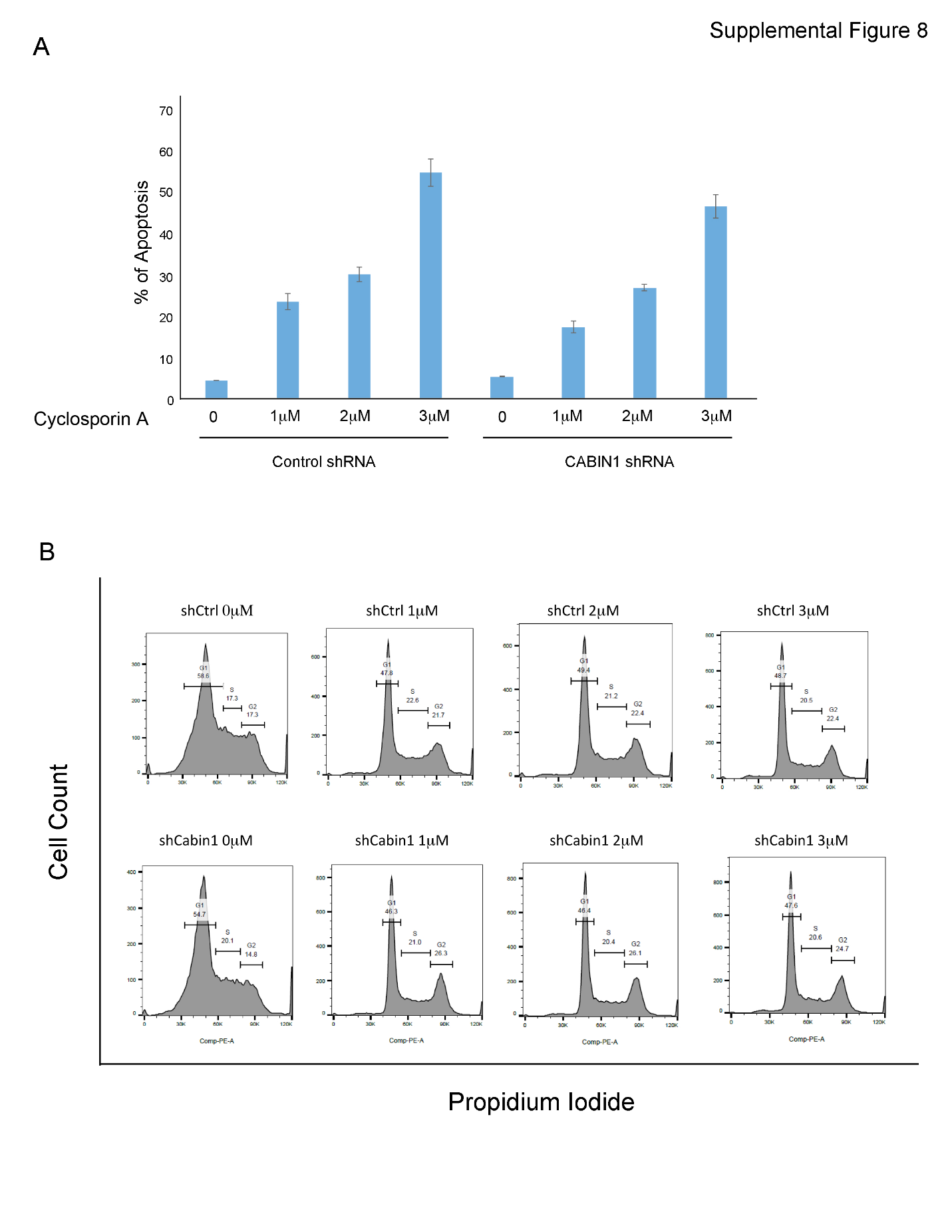


**Supplemental Figure 8. Effect of cyclosporin A on apoptosis and cell cycle in MZL cells.**

A). SSK41 WT and CABIN1 KD cells were treated with the indicated concentrations of cyclosporin A for 4 days and analyzed for apoptosis by flow cytometry. B) SSK41 WT and CABIN1 KD cells were treated with the indicated concentrations of cyclosporin A for 2 days and cell cycle analyzed by flow cytometry.
